# Costs of Rearing the Wrong Sex: Cross-Fostering to Manipulate Offspring Sex in Tammar Wallabies

**DOI:** 10.1371/journal.pone.0146011

**Published:** 2016-02-05

**Authors:** Lisa E. Schwanz, Kylie A. Robert

**Affiliations:** 1 School of Marine and Tropical Biology, James Cook University, Townsville, QLD, Australia; 2 Institute for Applied Ecology, University of Canberra, Canberra, ACT, Australia; 3 School of Animal Biology, The University of Western Australia, Perth, WA, Australia; 4 Department of Ecology, Environment and Evolution, La Trobe University, Bundoora, VIC, Australia; Universidad de Granada, SPAIN

## Abstract

Sex allocation theory assumes that offspring sex (son vs. daughter) has consequences for maternal fitness. The most compelling experiment to test this theory would involve manipulating offspring sex and measuring the fitness consequences of having the “wrong” sex. Unfortunately, the logistical challenges of such an experiment limit its application. In tammar wallabies (*Macropus eugenii*), previous evidence suggests that mothers in good body condition are more likely to produce sons compared to mothers in poor condition, in support of the Trivers-Willard Hypothesis (TW) of condition-dependent sex allocation. More recently, we have found in our population of tammar wallabies that females with seemingly poor access to resources (based on condition loss over the dry summer) are more likely to produce sons, consistent with predictions from the Local Resource Competition (LRC) hypothesis, which proposes that production of sons or daughters is driven by the level of potential competition between mothers and philopatric daughters. We conducted a cross-fostering experiment in free-ranging tammar wallabies to disassociate the effects of rearing and birthing offspring of each sex. This allowed us to test the prediction of the LRC hypothesis that rearing daughters reduces the future direct fitness of mothers post-weaning and the prediction of the TW hypothesis that rearing sons requires more energy during lactation. Overall, we found limited costs to the mother of rearing the “wrong” sex, with switching of offspring sex only reducing the likelihood of a mother having a pouch young the following year. Thus, we found some support for both hypotheses in that rearing an unexpected son or an unexpected daughter both lead to reduced future maternal fitness. The study suggests that there may be context-specific costs associated with rearing the “wrong” sex.

## Introduction

Does the production of a daughter versus a son influence maternal fitness? Offspring sex could be extremely important for maternal fitness, and empirical tests across living organisms have provided compelling confirmation of many hypotheses [1,2]. Trivers and Willard [3] suggested that, under certain life histories, maternal condition and offspring provisioning (embryonic or juvenile) would be more important for the fitness of sons due to higher variance in reproductive success among males compared to among females. Assuming offspring provisioning influences reproductive success more strongly in sons than in daughters, mothers of greater condition are predicted to produce more sons than those of poor condition (Trivers-Willard Hypothesis, TW; [4–7]). Different provisioning rates for sons and daughters may also have direct fitness consequences for mothers if sons ‘cost’ more to rear and diminish maternal body reserves for future reproduction [5,8](although this is not strictly true if mothers invest according to their optimal levels regardless of the offspring sex).

For species with extended parent-offspring interactions, additional cost or investment into philopatric offspring may be accrued once they are nutritionally independent from their mothers. Mothers may indirectly benefit from producing the philopatric sex if they can bequeath the benefit of a high quality habitat or high social rank (Local Resource Inheritance, LRI, or Rank Inheritance; [5,9–13]), and may directly benefit if philopatric offspring increase the future reproductive success of the mother through cooperation (Local Resource Enhancement, LRE; [14–18]). In contrast, mothers may benefit from producing an offspring of the dispersive sex if philopatric offspring would cause competition for access to limited local resources with the mother or with siblings (Local Resource Competition, LRC; [10,18–23]). Importantly, the TW and Local Resource (LRI/C) hypotheses of sex allocation may operate simultaneously in a given population if individual mothers differ in their ability to provision offspring or in their access to resources post-weaning [22–24].

Testing sex allocation predictions typically involves either: 1) examining correlations between offspring sex ratio and a proposed ecological, social or physiological factor (e.g. [4,13,16,21,22]), or 2) manipulating a proposed factor and examining the resultant offspring sex ratios (e.g. [25–28]). Unfortunately the most pertinent test of the fitness consequences of offspring sex is rarely attempted. Specifically, manipulation of offspring sex ratio itself via cross-fostering allows examination of the fitness consequences of rearing sons or daughters independent of any factors inherent to the birthing of each sex. [26,29–33]. This type of experiment is typically prohibitively difficult for sex allocation studies in birds and mammals due to 1) inability to know offspring sex early in development, 2) challenges of maternal acceptance of cross-fostered offspring, and 3) access to offspring prior to major investment by the mother.

In this study, we took advantage of marsupial reproductive biology to manipulate offspring sex via cross-fostering offspring in a free-ranging marsupial mammal, the Tammar wallaby (*Macropus eugenii*), in order to examine how rearing a son or daughter impacts immediate aspects of maternal fitness. This innovative approach to studying sex allocation is particularly powerful when applied to marsupials [34]. The highly altricial nature of marsupial neonates means that, at the time of cross-fostering, mothers have made nearly no energetic investment in their offspring (neonates are <0.5% of maternal mass; [35–37], so almost all energetic investment is made into the reared offspring following the cross-fostering. In macropods, offspring sex is readily identified by external features within days after birth (see methods), and mothers accept non-offspring pouch young [31,38]. Females have only one offspring at a time in their pouch, removing the possibility of confounding effects of litter size and offspring sex ratio [39,40]. Further, in the tammar wallaby, reproduction is highly seasonal and synchronized across a population, allowing size-matching of cross-fostered offspring and minimizing direct conflict between successive offspring.

Across studies of the tammar wallaby, there has been evidence that maternal investment during lactation (i.e. TW) as well as competition post-weaning (i.e LRC or LRI) are important correlates of offspring sex. Mothers of higher body condition have been recorded to be more likely to carry male pouch young [13,41], and there is evidence that mothers that birth a son commit more to lactation and weaning success regardless of which sex they rear, although mass at weaning does not differ between the sexes [31,42]. Moreover, dominance hierarchies among adult males are based on body size and strongly influence reproductive success [43–45]. These lines of evidence suggest a TW effect may be operating in this species.

More recently, we found that LRC or LRI also potentially operates in a high-density population of tammar wallaby [13]. Specifically, mothers that maintained their relative body condition over the driest part of the year (presumably with high relative resource consumption) were more likely to carry daughters in their pouch [13]–a previously-undocumented association between high resource access and the production of daughters in this species. In addition, several lines of evidence suggest post-weaning associations between mothers and daughters are important. Philopatry appears to be greater for females than males [46,47], and philopatric offspring often associate behaviourally with their mothers for several months after weaning (see also in grey kangaroos, *Macropus giganteus*; [48]). Moreover, female tammar wallabies have overlapping home ranges (~2–4 ha) in which they may forage in aggregations at dusk [49–51]. Thus, spatial and behavioural associations between female kin are likely and could lead to cooperative or competitive interactions [52–54].

**Fig 1:**
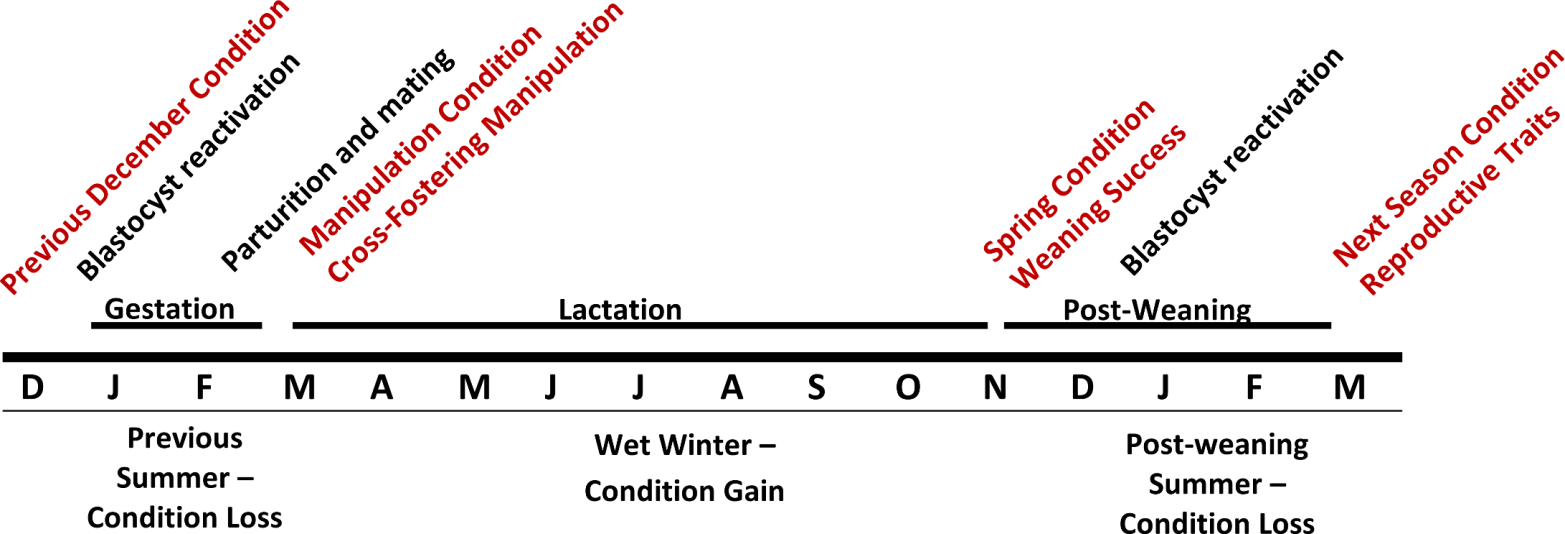
Timeline of study with respect to tammar wallaby reproduction. This study ran December 2009 to March 2011. We recorded maternal condition in the summer previous to our cross-fostering manipulation (prior to blastocyst reactivation). Then, pouch young were cross-fostered early in the pouch stage (March), and maternal outcomes were measured across lactation (December), across the post-weaning summer and into the following reproductive season (following March). Offspring manipulated in this study could have resulted from eggs fertilized between approximately one year and one month prior to birth.

Confusingly, although maintenance of body condition over the dry summer was associated with the production of daughters in both years of our study (consistent with LRC/LRI), higher body condition at the start of the summer (the time of embryonic reactivation; Fig 1) in one year (the second year) of our study was associated with producing sons (consistent with TW; [13]). This is because change in condition over the second summer was related to initial body condition–the highest-residual mothers in December had the greatest loss in condition by March [55]. With the goal of assessing whether LRC/LRI, TW or both operate in our study population, we performed a cross-fostering experiment to examine the direct fitness consequences of rearing the “wrong” sex.

If producing daughters bears a cost due to competition for local resources post-weaning (LRC or LRI), we predicted that rearing daughters compared to sons would be associated with poorer maintenance of body condition following weaning and poorer reproductive output the following year. According to this hypothesis, mothers that are better able to bear these costs (higher local resources available post-weaning) are more likely to birth daughters than sons, assuming predictability of resource availability post-weaning. Thus, we predicted the poorest post-weaning outcomes for mothers that birthed sons and reared daughters. In contrast, if mothers invest more into sons than daughters (TW hypothesis), we predicted that rearing sons compared to daughters would be associated with reduced gain in body condition during lactation, altered weaning success, and potentially poorer reproductive output the next year. If mothers that birth sons are more capable of making this investment, we predicted the poorest weaning outcomes for mothers that birthed daughters and reared sons.

## Materials and Methods

### Study animal and site

All work was conducted under the ethical approval of University of Western Australia’s Animal Ethics Committee (approval: RA/3/100/897) and with the permission of the Australian Department of Defence. Tammar wallabies (*Macropus eugenii*) are macropod marsupials native to Australia. They are relatively small-bodied (adult females 3–5 kg), herbivorous, and moderately social at high-densities. We trapped tammar wallabies (*Macropus eugenii*) on Garden Island (115°40’E 32°16’S), 5 km off the coast of Western Australia. We focused our cross-fostering experiment on wallabies found on the navy base (Fleet Base West, Australian Department of Defence), which occupies ~25% of the island. In this subpopulation, the wallabies live at high densities and have access to supplemental food and water (irrigated lawns and sports fields; see [55]).

Tammar wallabies are seasonal, highly-synchronous breeders, with most females giving birth to a single, highly altricial neonate in the dry austral summer (January-March, median birthdate February 20th; [13,51,56,57] see Fig 1). Peak lactation occurs over the relatively wet austral winter when food resources are highest, and the pouch young are fully weaned in spring, when approximately 270–350 days old (November-December; [56]). Birth each summer is followed by a post-partum estrus and diapause of the resulting embryo until the following summer solstice or if lactation of the pouch young terminates prior to June (i.e. pouch young death; [56]). Thus, unlike many macropods, temporal overlap of offspring is limited to one lactating offspring and one diapaused embryo.

Despite the energetic investment of lactation, most females gain body condition during the winter [13,55] and rely on current nutrition rather than stored reserves to meet lactational demands [36]. Body condition is lost over the summer when annual precipitation is at its minimum [55]. During this time, offspring are recently-weaned and reproductive females undergo a ~30-day gestation of the subsequent reactivated embryo ([56]; Fig 1). Together, these life histories suggest that among-individual variation in resource access and ability to provision offspring should be described better by change in condition during the driest part of the year than by maternal body condition at blastocyst reactivation. Mechanistically, manipulation of offspring sex could occur at initial fertilization nearly one year prior to birth (and 1.5–2 years prior to lactation and post-weaning interactions), or via selective blastocyst abortion following reactivation [6,58] and re-fertilization of a new blastocyst one month prior to birth.

### Study methodology

We trapped wallabies using soft-wall Thomas traps (450 × 450 × 800 mm, Sheffield Wire Products, Welshpool, WA, Australia) baited with ‘kangaroo muesli’ and set just before dusk. All adult animals were given ear tags for individual identification (National Band and Tag model Jiffy 893), and measured for mass, foot (pes) length, and reproductive status (pouch young present/absent, teat elongated/regressed; [55]). We initiated our cross-fostering experiment in March 2010 (Fig 1), when most females had a small, furless pouch young permanently attached to the teat. We recorded the presence, sex and head length of the pouch young (PY Size; proxy for PY age). Sex can be observed 5–10 days after birth by the presence of a scrotal sac on males and a pouch indentation on females. Head length of pouch young was measured with digital calipers (to 0.01 mm). Females with pouch young too young to measure or identify sex (approx. < 8 days old) were not used in the experiment. At the time of cross-fostering, pouch young of mothers used in the experiment averaged 41.4 ± 12.8 days old (range 8–72 based on head length growth tables, [59]; up to approximately 30g or <1% of maternal body mass; [35]). At this age, maternal energetic investment in offspring has been negligible [35–37]. As described above, females that had maintained their body condition over the previous summer (Dec–Mar; Fig 1) were more likely to have a daughter in their pouch at the time of cross-fostering than were females that had decreased in body condition [13], although this did not mean they were in the best condition at cross-fostering (see results). Maternal body condition at blastocyst reactivation (previous December) or at the time of manipulation was not correlated with sex of the pouch young ([13] and unpublished results).

Seventy females (32 carrying daughters, 38 carrying sons) were used for the cross-fostering experiment, and assigned to one of four treatments based on the sex birthed and the sex reared by the mother (birthed/reared): 1) Female/Female, *N* = 18; 2) Female/Male, *N* = 14; 3) Male/Female, *N* = 14; 4) Male/Male, *N* = 24. Included in these sample sizes are 14 mothers that reared their own offspring following brief removal from the teat (sham manipulation, 8 males and 6 females). Because weaning success of sham manipulated individuals did not differ from same-sex cross-fostered (cross-fostered, 82.4% (14 of 17 mothers retrapped in the spring); sham manipulated, 75.0% (6 of 8 mothers retrapped in the spring); *G* (cross-fostering) = 0.19, *p* = 0.66), we combined them in the F/F and M/M treatments for all analyses. Experimental females were sedated five minutes prior to cross-fostering with a mixture of Ketamine (10mg/kg) and Xylazine (1.25mg/kg) injected intramuscularly. This provided light anesthesia for up to one hour. Cross-fostering was performed using established protocol by gently pulling the teat out of the pouch young’s mouth and re-attaching with forceps [31,38]. Young were micro-chipped (12 mm x 2.1 mm ISO FDX-B transponder, Allflex, Capalaba, Queensland, Australia) and re-attached to the foster mother’s teat. All cross-fostered offspring pairs were of equivalent developmental stages to avoid problems due to changes in milk composition over the course of lactation that impacts growth rate [60,61]. Thus, assignment of mothers into each treatment group was based on the availability of appropriate pairs trapped on the same night.

To record the impact of cross-fostering treatment on experimental females, we trapped the population again at the end of lactation (late November to early-December 2010), and during the early pouch young stage of the following year (March 2011; Fig 1). We recorded weaning success (mothers weaned their offspring if their teats were still elongated and their mammary gland regressing in December; [55]), and presence and sex of a pouch young the following year. Maternal body condition was calculated as the residuals of a linear regression of body mass vs. pes length of all parous females trapped in the focal population and a nearby natural bush population across four sessions of trapping (December 2009/10, March 2010/11; see [55] for regression statistics; S1C Table). The residuals, therefore, reflect a broad view of how body condition compares across seasons (being higher in December vs. March) and natural vs. naval base populations (being higher on the naval base; [55]). This cross-seasonal approach allows estimating absolute loss in body condition over the summer and gain in body condition over the winter (rather than relative change). Thus, using these residuals, we calculated changes in body condition during lactation (March 2010 –December 2010) and over the summer post-weaning (December 2010 –March 2011). Data are available in the S1 Table.

### Data Analysis

We examined the influence of offspring sex on maternal response variables by entering ‘birthed offspring sex’, ‘reared offspring sex’ and their interaction as predictors. If investment of energy during lactation into sons (i.e. TW Hypothesis) was an important cost for mothers during our study, we expected the predictors to be important in explaining weaning success or maternal condition change over lactation. If competition with daughters post-weaning (i.e. LRC/LRI Hypotheses) was important during our study, we expected the predictor variables to be significant in explaining variation in maternal condition change over the post-weaning summer and maternal reproductive traits the following year. For these post-weaning effects, we excluded from analyses mothers that had failed to wean their pouch young because we were only interested in those mothers with weaned offspring as potential competitors. For analyses of pouch young sex the following year, we excluded three mothers with pouch young too small to identify sex. Most of the response variables are also predicted by variation in maternal condition, so we entered a measure of maternal condition into the models. Because we found that maternal condition at the time of the manipulation varied among our treatments (see results), we chose this variable as our maternal condition covariate for all models (maternal condition is highly correlated across trapping seasons; [55]).

For the continuous response variables (condition, condition change and PY size), significance of predictor variables was assessed using p-values from simple univariate linear models. Change in condition during the summer post-weaning was not normally distributed, so we ran additional models with this response variable transformed using two separate methods (inverse and rank). The results did not differ qualitatively from the results from the untransformed data. For the categorical response variables (weaning success, and re-trapping, reproductive state and PY sex next year), we fit models using the ‘Generalized Linear Model’ personality in JMP (distribution = binomial; link function = logit) and assessed the significance of each predictor variable by comparing model fit (deviance) with and without the focal variable. The likelihood ratio statistic (G; difference in deviance of the two models) was compared to a χ^2^ distribution, with DF = 1 for simple predictors and DF = 2 for the interaction term. AICc of each model were additionally compared to note whenever AICc from competing models differed by less than 2.

## Results

Despite efforts to randomize treatments, maternal body condition at the time of cross-fostering differed among treatments, with those mothers that birthed *and* reared sons having a much higher relative body condition than the three other treatments (Table 1; LSM ± SE, F/F:-33.47 ± 88.93; F/M:-141.58 ± 100.83; M/F: -137.21 ± 104.64; M/M:150.89 ± 78.67). Head length, and therefore age, of pouch young at cross-fostering did not differ among treatments (Table 1). The likelihood of retrapping an experimental female the following March (a proxy for survival) did not differ significantly among treatments (Table 1; F/F, 61.1%; F/M, 50.0%; M/F, 53.8%; M/M, 34.8%; overall retrapping success of 48.5% (33 of 68)).

**Table 1:** Influence of birthed and reared offspring sex on measures of direct maternal fitness in tammar wallabies. “Manipulation condition” was maternal residual body condition at the time of experimental cross-fostering. PY = Pouch Young. Sample sizes refer to total sample included in the analysis.

Variable	Predictors			
	birthed sex	reared sex	birthed sex *reared sex	manipulation condition
manipulation condition	F = 1.01	F = 0.92	**F = 4.46**	NA
*N* = 68^1^	p = 0.32	p = 0.34	**p = 0.04**	
(F/F,18; F/M,14; M/F,13; M/M,23)				
manipulation PY head length	F = 0.05	F = 0.06	F = 2.00	NA
*N* = 70	p = 0.83	p = 0.80	p = 0.16	
(F/F,18; F/M,14; M/F,14; M/M,24)				
lactation condition change	F = 0.05	F = 0.02	F = 0.36	**F = 4.95**
*N* = 41	p = 0.82	p = 0.90	p = 0.55	**p = 0.03**
(F/F,13; F/M,7; M/F,9; M/M,12)				
weaning success	G = 0.00	G = 0.32	G = 0.01	G = 0.12
*N* = 41	p = 1.00	p = 0.57	p = 1	p = 0.73
(F/F,13; F/M,7; M/F,9; M/M,12)				
next year re-trapping success	G = 1.45	G = 1.52	G = 0.11	G = 0.02
*N* = 68	p = 0.23	p = 0.22	p = 0.95	p = 0.89
(F/F,18; F/M, 14; M/F, 13; M/M,23)				
post-weaning condition change^2^	F = 2.00	F = 0.03	F = 0.02	F = 0.48
*N* = 21	p = 0.18	p = 0.87	p = 0.88	p = 0.50
(F/F,8; F/M,4; M/F,4; M/M,5)				
presence of PY next year^2^	G = 0.00	G = 0.00	**G = 10.82**	**G = 6.03**
*N* = 27	p = 1	p = 1	**p = 0.005**	**p = 0.01**
(F/F,9; F/M,6; M/F,6; M/M,6)				
PY sex next year^3^	**G = 5.73**	**G = 4.95**	G = 0.45	**G = 9.81**
*N* = 22	**p = 0.02**	**p = 0.03**	p = 0.80	**p = 0.002**
(F/F,9; F/M,4; M/F,3; M/M,6)				

^1^Two females had improper measures of mass or pes length, thus could not be used in residual calculations.

^2^Excluded 8 mothers that failed to wean their offspring.

^3^Excluded 3 mothers with offspring too small to identify sex.

**Fig 2:**
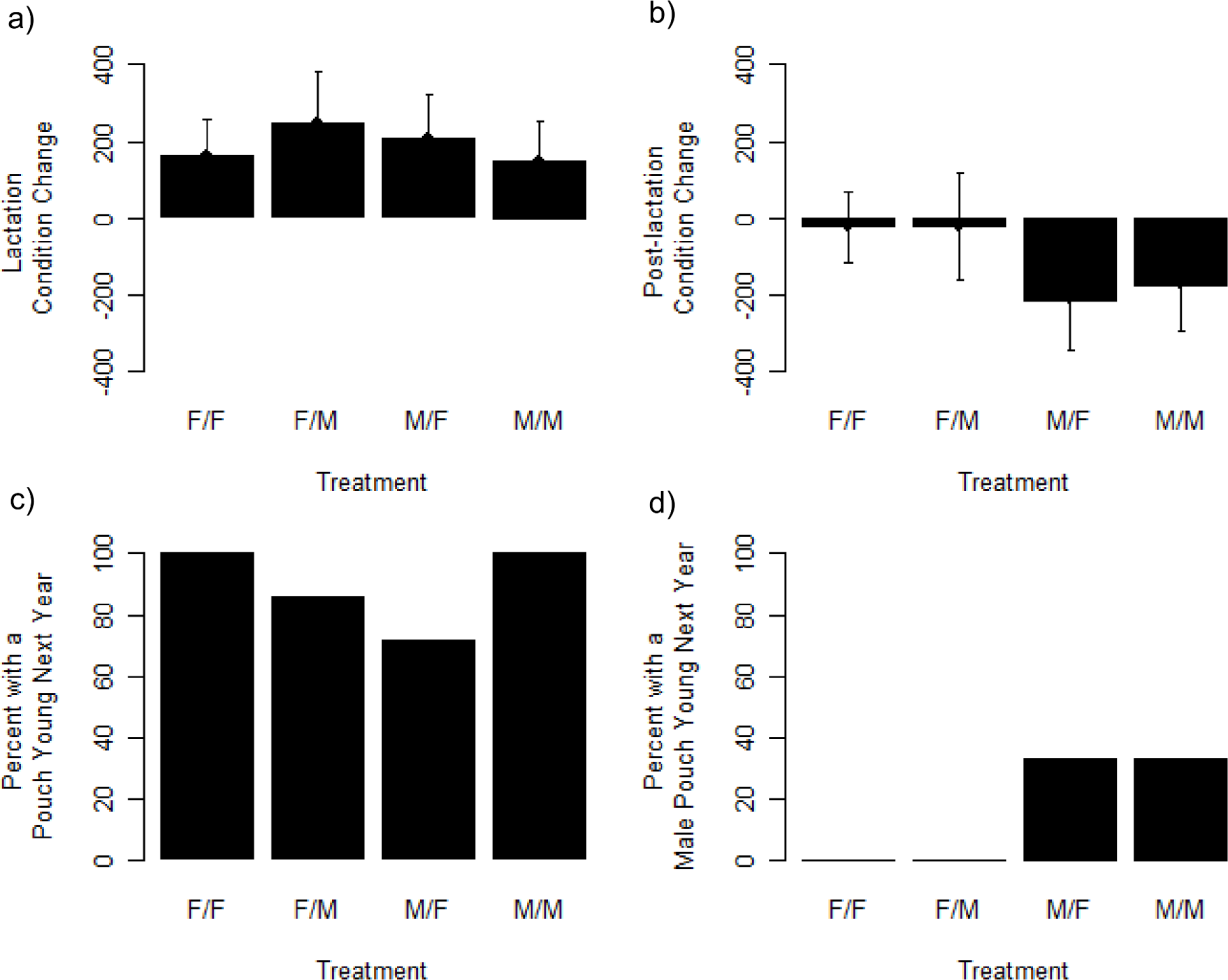
Effects of cross-fostering offspring sex on maternal body condition and subsequent reproduction in tammar wallabies. Mothers gain (residual) body condition during lactation (a), which coincides with the season of greatest resources, and lose body condition post-lactation (b) during the dry summers. Values in (a) and (b) are presented as Least Square Means ± SE from the statistical model. The percent of mothers with pouch young early in the following reproductive season (c) was influenced by the cross-fostering treatment, whereas the sex of the pouch young during the following season varied strongly depending on the sex birthed during the experimental year (d). Treatments indicate ‘Birthed Sex / Reared Sex’ of offspring, with F = female and M = male.

Change in body condition over lactation was negatively correlated with initial condition (parameter estimate = -0.32), but did not differ among our cross-fostering treatments (Table 1; Fig 2A). Similarly, weaning success was generally quite high (overall 80.4%; F/F, Weaned (Total): 10 (13); F/M, 6 (7); M/F, 7 (9); M/M, 10 (12)) and did not differ according to treatment. Thus, our data provided no support for energetic costs during lactation being associated with offspring sex (TW).

When considering the effects of cross-fostering on females after successful weaning, we found that reproduction the following year was influenced by treatment. Females that reared the sex opposite of what they had birthed were less likely to have a pouch young the following March compared to those that reared the same sex they birthed (Table 1; Fig 2C; F/F, Pouch young present (Total), 9 (9); F/M, 5 (6); M/F, 4 (6); M/M, 6 (6)). While accounting for offspring sex effects, mothers that had better body condition at the start of the experiment were less likely to have a pouch young the following year (parameter estimate = -0.01 for log odds of PY/no PY; Table 1). The reproductive differences among treatments in the following year were not explained by loss of condition post-weaning (Table 1; Fig 2B). Of the eight females known to have failed at weaning their offspring, six were retrapped in the spring and all had a pouch young (2 F/F, 1 F/M, 1 M/F, 2 M/M), which is consistent with costs of reproduction being associated with post-weaning effects.

In addition, we saw a correlation between birthed offspring sex between the two reproductive years, despite very low sample sizes in some of our treatment groups (Table 1; Fig 2D). All females that birthed daughters in the year of our cross-fostering also birthed daughters the following year, regardless of the sex of offspring they reared. Females that had birthed sons in the first year of study produced 33% sons the following year. Reared sex was also significant at the α = 0.05 level, and its inclusion produced the model with the lowest AICc score (12.45). However, the data do not appear supportive of an effect of reared sex (there is no difference between F/F and F/M or between M/F and M/M), and the next-best model without reared sex had a δAICc < 2 (AIC = 14.39), thus the model without reared sex should be favored. Mothers that had better body condition at the start of the experiment, when they were likely conceiving next year’s embryo, were more likely to have a son the following year (parameter estimate = 0.33 for log odds of Male/Female; Table 1). The same sex ratio trend was seen in females that did not successfully wean their offspring, with the three M/F or M/M mothers having sons the following year, and the three F/F and F/M mothers carrying two daughters and one offspring of indistinguishable sex.

## Discussion

In this study, we tested the importance of offspring sex (male or female) for immediate components of maternal fitness by performing a logistically-challenging cross-fostering experiment in a free-ranging wild population of marsupial mammals. We leveraged the highly altricial nature of marsupial neonates to cross-foster prior to nearly all maternal investment.

Overall, we found little impact of rearing male or female offspring on maternal fitness components in tammar wallabies. Our cross-fostering treatment impacted only a mother’s reproductive status in the year following the manipulation. Specifically, the sex of the offspring reared impacted the likelihood that a mother had a pouch young the following year, but this effect differed depending on the sex birthed. Mothers that birthed daughters saw a more negative effect from rearing sons, while mothers that birthed sons saw a more negative effect from rearing daughters. Broadly, these results suggest that rearing the “wrong” sex is costly, rather than one sex of offspring being uniformly more or less costly.

These results provide further evidence for our previous interpretations of sex ratios in tammar wallabies–namely, that offspring sex ratio patterns do not fit neatly within a single hypothetical framework such as LRC or TW. If birthing a son but weaning a daughter leads to a delay or prevention of reproduction the following reproductive season due to having an unanticipated daughter nearby, the results are consistent with the LRC hypothesis.

In contrast, costs accrued when rearing an unanticipated son is consistent with the TW hypothesis. The idea that individual mothers within a population experience different types of costs from sons or daughters (e.g. both TW and LRC apply) is supported by evidence in other taxa that maternal age, maternal reproductive traits, population density, and the environment can influence the patterns in sex ratio bias [8,24,32,62–64]. For example, the tendency for the impact of philopatric offspring to vary among parents based on local resource access has been documented in birds and mammals [11,16,22,24].

In our tammar wallaby population, it is difficult to untangle the importance of maternal local resource availability (e.g. LRC) and maternal body condition (e.g. TW) for offspring sex [13]. Females in the present study that had birthed daughters were those that maintained their body condition over the summer better than those with sons [13], whereas in the reproductive year following our manipulation, higher maternal condition at blastocyst reactivation was associated with the production of sons. The reduced reproduction in female wallabies that reared the “wrong” sex suggests that mothers vary in the extent to which they can afford the costs of lactating or interacting post-weaning with sons and daughters. Local habitats within a female’s home range may differ according to whether they provide high food or shelter during the wet-season/lactation or dry-season/post-weaning. Alternatively, maternal age may play a large role in determining costs of sons and daughters [8,24]. Unfortunately, we do not have reliable age data from our population to examine whether young mothers (with several future years of interaction with philopatric daughters) tended to overproduce sons. In sum, the results indicate that sex allocation strategies are best examined on an individual basis rather than assuming the same pattern applies to all individuals. If a mother typically produces the sex of offspring with the lower cost given her individual situation, then rearing the “wrong” sex would be costly regardless of which sex she birthed.

This explanation must be tempered by the fact that the statistically-significant results of future reproductive success are based on a modest sample size. Limitations on sample size are an unavoidable product of conducting such a challenging experiment on wild mammals. Data on post-weaning maternal traits represent only 30–40% of the females assigned to the study due to incomplete recapture. This trapping bias, however, could not have introduced erroneous treatment results because re-trapping of females the following year was not influenced by our cross-fostering treatments.

Future examination of the costs of offspring sex in this system should involve more direct quantifications of maternal resources access and describing sex-specific, mother-offspring interactions post-weaning. We focused on change in body condition when competition for food resources is at its most intense (the dry summer) as an indicator of relative resource access because it varies substantially among females and has been demonstrated to be related to offspring sex [13]. However, while maternal morphological body condition is often used in sex allocation studies, it is a poorer predictor of offspring sex compared to behavioural indicators or direct measures of resources access [6,65]. Moreover, body condition can be problematic due to interpreting correlations between initial condition and change in condition and because it can reflect relevant differences in age [8,66]. Using physiological or behavioural measures of resource consumption (e.g. [67–69], or quantifying non-food resources important for reproduction [22] may provide additional insight into sex allocation, competition and costs of reproduction in this study system.

The results from the present cross-fostering study contrast to those from a previous cross-fostering study we performed on tammar wallabies being translocated from a different wild population to a captive setting [31]. In the previous study, weaning success was overall much lower, and was particularly low for mothers that had birthed daughters. The interpretation of this result was that mothers that birth sons commit early during reproduction to a greater level or persistence of investment, consistent with the TW hypothesis. In this current study, weaning success did not depend on offspring sex (birthed or weaned), and we did not find overarching support for the TW hypothesis. The disparity in the two studies could be attributed to population differences, or to the stress of relocation [70]. One speculative possibility is that relocation to high density conditions triggered sudden abandonment of potentially philopatric daughters (assuming the mothers had no knowledge of the sex switch).

Finally, we found an unanticipated and intriguing result when considering the sex of the pouch young the following year. In particular, all 13 mothers that birthed a daughter during the year of our manipulation and were retrapped the following year also birthed a daughter the following year, regardless of which sex they reared. In contrast, the nine mothers that had initially birthed sons and were retrapped showed more equal production of sons and daughters the following year. This was associated with a dramatic difference in the overall offspring sex ratio between the two years across our population – 59% sons in 2010 and 38% sons in 2011 [13]. This interannual pattern is clearly not explained solely by the relative costs or benefits of rearing each sex, given that it was apparent regardless of our cross-fostering manipulation. Mothers may practice among-year strategies of sex allocation (potentially linked to individual ‘quality’). A markedly different pattern of sex-ratio trade-offs between consecutive litters occurs in the bank vole, where the sex ratio of the litter being lactated and the sex ratio of the litter in utero interact to determine maternal energetic costs [33].

In common with the few other cross-fostering studies of offspring sex ratio [30–33], the costs of offspring sex to mothers in our study provides insight as well as more questions. Similar to findings in great tits (*Parus major*; [32]), the costs of offspring sex to female tammar wallabies were more modest than we predicted. In addition, as more cross-fostering studies accumulate, it becomes clearer that the costs of sons and daughters are not fixed, but context-dependent and variable among mothers [30,31,33].

## Supporting Information

S1 Table Supporting data for Tammar wallaby cross-fostering study. Table A, Cross-fostering experiment: An excel spreadsheet of data from the cross-fostering experiment, including offspring and maternal traits. Table B, Experimental metadata: An excel spreadsheet explaining column meanings. Table C, Raw mass and size data: An excel spreadsheet of mass and pes data collected from reproductive females trapped December 2009 –March 2011 used to calculate residual mass (condition) values.
(XLSX)
